# Air pollution, maternal hypertensive disorders, and preterm birth

**DOI:** 10.1097/EE9.0000000000000062

**Published:** 2019-08-29

**Authors:** Kari A. Weber, Wei Yang, Frederick Lurmann, S. Katharine Hammond, Gary M. Shaw, Amy M. Padula

**Affiliations:** a Department of Pediatrics, Division of Neonatal and Developmental Medicine, Stanford University School of Medicine, Stanford, California; b Sonoma Technology, Inc., Petaluma, California; c Environmental Health Sciences Division, School of Public Health, University of California, Berkeley, California; d Department of Obstetrics, Gynecology and Reproductive Sciences, University of California, San Francisco, California.

## Abstract

**Background:**

Air pollution has been associated with hypertension and preterm birth. We examined if prenatal exposure to air pollutants was associated with gestational hypertension and if its association with preterm birth was modified by maternal hypertension.

**Methods:**

Data were from birth certificates and hospital discharge records of 252,205 women in San Joaquin Valley of California from 2000 to 2006. Air quality data were assigned from 24-hour averages of nitrogen dioxide (NO_2_), particulate matter <10µm (PM_10_) and <2.5µm (PM_2.5_), and carbon monoxide (CO) for different averaging periods over pregnancy. We estimated odds of preterm birth and multiplicative interaction between each pollutant and hypertensive disorder.

**Results:**

Among normotensive women, odds of preterm birth were slightly higher for higher exposure to all pollutants over the entire pregnancy. Patterns were similar among women with a hypertensive disorder. Among 32–36 week births, there was effect modification for exposure to NO_2_ and CO during the first trimester with higher odds among hypertensive women, and PM_2.5_ and CO during the last 6 weeks with higher odds among normotensive women. For 28–31 week births, there was effect modification by hypertensive status for PM_10_ exposure for the entire pregnancy, first, and second trimester with hypertensive women consistently having lower odds of preterm birth than normotensive.

**Conclusion:**

There was some evidence of effect modification in the direction counter to our hypothesis for exposure to PM_10_ and early preterm birth, and CO and PM_2.5_ at the end of pregnancy, but overall, hypertension did not modify the relationship between pollution and preterm birth.

## Abstract 2

Supplemental Digital Content is available in the text.

**What this study adds:**

How air pollution influences risk of preterm birth has not been fully elucidated. We explored one of the primary indications of preterm birth, hypertension in women. To fill a knowledge gap regarding potential differences in the effect of air pollution on preterm birth in pregnant women with a hypertensive disorder compared with those without, we estimated potential effect modification using data from a highly polluted region of California.

## Introduction

Exposure to ambient air pollution, specifically particulate matter, has been associated with increases in blood pressure and hypertension in previous studies.^1,2^ A meta-analysis of exposure to ambient air pollution and pregnancy-induced hypertension also reported increased risks for particulate matter and nitrogen dioxide (NO_2_).^3^ While there has been some heterogeneity in the results of these studies, especially between different pollutants and timing of exposure, air pollution is believed to cause endothelial dysfunction and increase blood pressure through autonomic nervous system imbalance, oxidative stress, and systemic inflammatory response,^4–6^ thus making this relationship mechanistically feasible.

Ambient air pollution exposure during pregnancy has also been associated with preterm birth^7^ and a previous analysis of our cohort observed associations between increased exposure to particulate matter [particulate matter <10µm (PM_10_)] and [particulate matter <2.5µm (PM_2.5_)] and preterm birth, with the strongest associations observed for exposure during the second trimester.^8^ Hypertensive disorders have also been associated with preterm birth. Preeclampsia, a hypertensive condition affecting pregnancy, contributes substantially to preterm birth,^9^ and chronic hypertension has been associated with both spontaneous^10^ and medically-indicated preterm birth.^11,12^

Thus, using data from a highly polluted area of California, we sought to determine if the relationship between exposure to air pollutants [carbon monoxide (CO), NO_2_, PM_10_, PM_2.5_, and traffic density] and preterm birth may be modified by the presence of a hypertensive disorder. We examined possible effect modification by hypertensive status, separately by timing of preterm birth and each pollutant. We also assessed the potential for pregnancy-induced hypertension to mediate any relationship between air pollution and preterm birth.

## Methods

### Study population

Data were from 329,650 births to women living in four counties in the San Joaquin Valley of California (Fresno, Kern, Stanislaus, and San Joaquin) between 2000 and 2006. Birth certificate data from the California Department of Public Health were linked with the Office of Statewide Health Planning and Development California maternal and infant discharge data. Linkage was successful for 98.6% of records. Eligible births were singletons, had a gestational age within 20–42 weeks, a birth weight within 500–5,000 grams, and were not missing state file numbers. Air pollution assignments were 80% complete for CO, 94% for NO_2_, 93% for PM_10_, 93% for PM_2.5_, and 96% for traffic density resulting in a sample of 258,522 births. Subjects missing data on eligibility criteria, last menstrual period (used to determine gestational age at birth), or covariates used for adjustment (maternal age, race/ethnicity, education, prenatal care, and insurance type) were excluded. The final analytical sample consisted of 252,205 births.

Street addresses for maternal residence at birth were obtained from birth certificates and geocoded using ArcGIS software (ESRI, Redlands, California) and corrected using ZP4 software (Semaphore Corporation, Aptos, California). Air pollution exposure assessment has been previously described in detail elsewhere.^8^ Briefly, ambient air quality data were obtained from U.S. Environmental Protection Agency’s Air Quality System database (https://aqs.epa.gov/aqsweb/documents/data_mart_welcome.html). Data were collected for daily 24-hour averages of (NO_2_, PM_10_, PM_2.5_, CO). Up to four air quality measurement stations were used to interpolate air quality with inverse distance-squared weighting. A maximum interpolation radius of 25 km was used for NO and CO_2_ and 50 km for PM_10_ and PM_2.5_. Values for residences located within 5 km of 1 or more monitoring stations were interpolated based on those stations only. Traffic-density measures were also collected based on distance-decayed annual average daily traffic volumes from the Geographic Data Technology traffic count data. Averages were calculated for each trimester, the entire pregnancy, and the last 6 weeks of pregnancy.

Additional maternal covariates included were age (<20, 20–24, 25–29, 30–34, ≥35 years), maternal race/ethnicity (White nonHispanic, Asian, African American, Hispanic, other), education (some high school or less, high school diploma, some college, college graduate, or more), parity (0, >1), prenatal care (initiated in first trimester), Medi-Cal (Medicaid) or other government program payment of birth costs, and infant sex. A previously created indicator variable for low neighborhood socioeconomic status (unemployment >10%, income from public assistance >15%, and families below poverty level >20% in the 2000 US Census at the block group level) was also included.^8^ This study was approved by the California State Committee for the Protection of Human Subjects and the Institutional Review Boards of Stanford University and the University of California, Berkeley.

### Statistical analysis

Preterm birth was defined as gestational age <37 weeks and was further divided into categories (20–27, 28–31, 32–33, 34–36 weeks). Distribution of maternal covariates were examined by gestational ages of infants’ birth. Each pollutant was divided into quartiles and the highest quartile was compared with the lower three quartiles combined. Hypertensive disorders were obtained from OSHPD using ICD-9 Codes. For the first analysis, pregnancy-induced hypertension [gestational hypertension and preeclampsia (642.3–642.7)] was the main outcome. Timing of pollutant exposure did not include end of pregnancy in an attempt to account for temporality due to general diagnosis of pregnancy-induced hypertension earlier in pregnancy. An ad hoc indirect effects mediation analysis was then performed for a particular pollutant if an association was observed. For the second analysis, chronic hypertension (401–405, 642.0–642.2, 642.9) was also included. For timing of exposure in the latter analysis, we additionally included the last 6 weeks of pregnancy to account for differences in gestational age and thus differing third trimester lengths and averaging periods between term and preterm births. Logistic regression was performed to estimate odds ratios (OR) and 95% confidence intervals (95% CI) and we considered a 20% higher odds to be “meaningful.” We stratified by the presence of a hypertensive disorder to test for effect modification of the association between each pollutant and preterm birth. We also tested for multiplicative interaction using a Wald test.

All analyses were adjusted for covariates believed to be confounders (maternal age, race/ethnicity, education, insurance type, and prenatal care). We also performed additional sensitivity analyses, one restricting to spontaneous preterm birth (preterm labor or premature rupture of membranes), one additionally adjusting for season of conception, and one excluding women with diabetes. All analyses were performed using SAS version 9.4 (SAS Institute, Cary, NC).

## Results

Early preterm births (gestational age ≤31 weeks) were more likely among women who were under age 20, Black non-Hispanic, and high school educated. Preterm births were more likely among women with medical care funded by Medi-Cal, who did not receive prenatal care in the first trimester and who were of low neighborhood socioeconomic status compared with term births. Women with pregnancy-induced hypertensive disorders were more likely to deliver preterm and those with preexisting hypertension were slightly more likely to deliver preterm compared with normotensive women (Table 1).

**Table 1:**
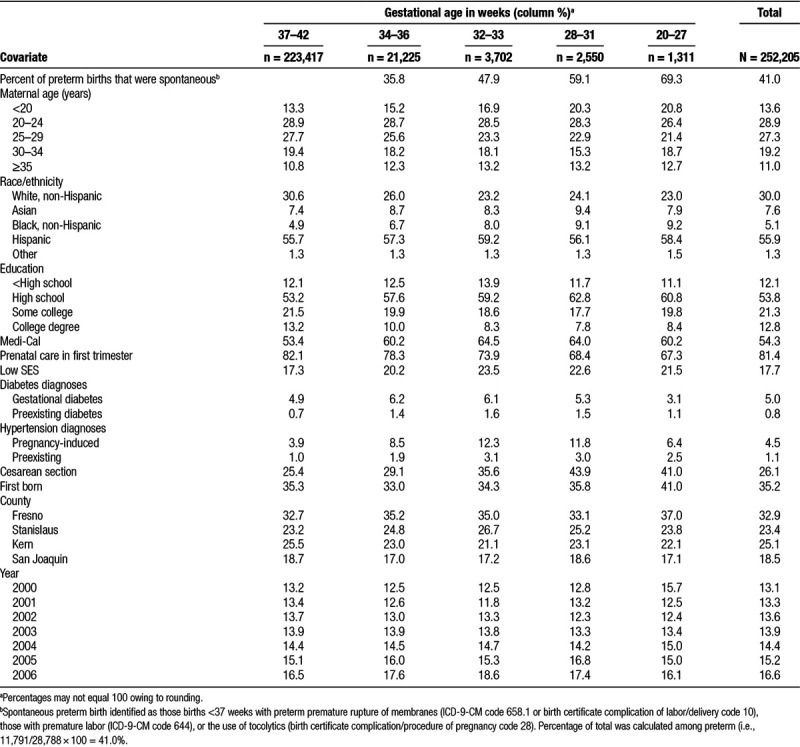
Distribution of Covariates by Gestational Age in Births in the Four Most Populous Counties in San Joaquin Valley, California, 2000–2006

The pollutants were highly correlated with each other. The Pearson correlations were 0.75, 0.57, and 0.76 between CO and NO_2_, PM_10_, and PM_2.5_, respectively, 0.49 and 0.59 between NO_2_ and PM_10_ and PM_2.5_, respectively, and 0.70 between PM_10_ and PM_2.5_ (results not shown). Correlations between the pollutants and traffic density were low. The associations between the individual air pollutants and pregnancy-induced hypertension are shown in Table 2. For exposure averaged over the entire pregnancy, there was a slight inverse association between exposure to PM_10_ and pregnancy-induced hypertension comparing the highest quartile of exposure to the lower three quartiles. There was no association when restricted to exposure in the first or second trimesters. There was a suggestive association between exposure to PM_2.5_ and pregnancy-induced hypertension for exposure averaged over the entire pregnancy and for exposure in the highest quartile during the second trimester. Associations for the other air pollutants and exposure periods were null. The indirect effects analysis resulted in a β ~ 0 (results not shown).

**Table 2:**
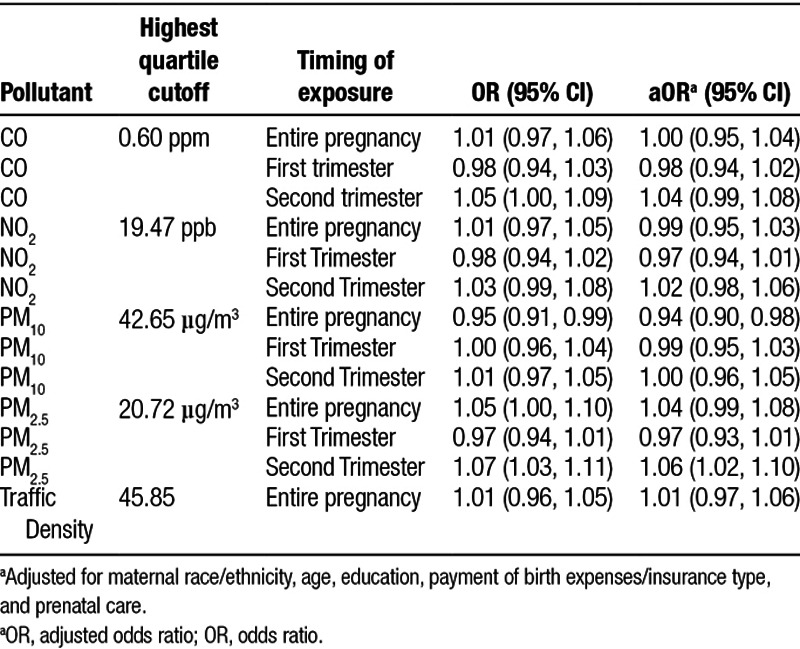
Association Between Air Pollutants and Pregnancy-Induced Hypertension, Comparing the Highest with the Lower Three Quartiles, by Timing of Exposure, 2000–2006

Associations between each air pollutant and preterm birth, stratified by presence of a maternal hypertensive disorder and gestational age, are presented in Table 3. For later preterm births (32–36 weeks gestation), among women without a hypertensive disorder, there were slightly higher odds for higher exposure over the entire pregnancy to all pollutants. Among these later preterm births, there were higher odds (i.e., ≥20%) for PM_2.5_. Patterns were similar for women with a hypertensive disorder.

**Table 3:**
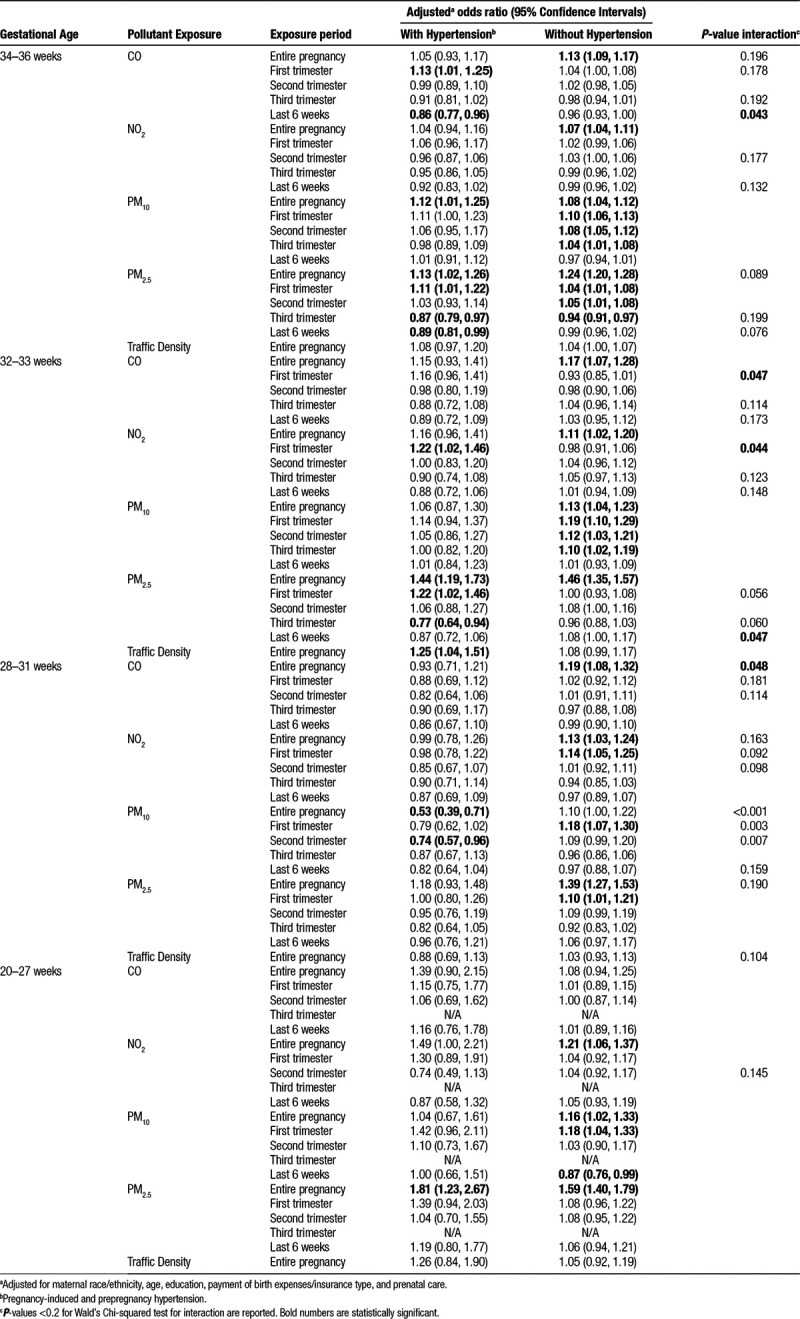
Associations Between Air Pollutants and Preterm Birth by Gestational Age and Timing of Exposure, and Effect Modification by the Presence of Maternal Hypertension, 2000–2006

For preterm births (32–33 weeks), results for first trimester exposures were slightly different among women with a hypertensive disorder. There were higher odds for exposure to NO_2_ among women with a hypertensive disorder, for which there was significant effect modification (*P* = 0.04), and PM_2.5_, for which the significance test for effect modification approached significance (*P* = 0.06).

In the later preterm births, we also observed effect modification for exposure to CO and PM_2.5_ during the last 6 weeks of pregnancy and CO during the first trimester. However, patterns were inconsistent by exposure timing. Odds of preterm birth for exposure to CO were higher among hypertensive women than normotensive women for exposure during the first trimester and lower among hypertensive women for exposure during the last 6 weeks.

For preterm births between 28 and 31 weeks gestation, among women without a hypertensive disorder, there were also slightly higher odds for higher exposure over the entire pregnancy to all pollutants (eTables; http://links.lww.com/EE/A56). Patterns were not the same among women with a hypertensive disorder except for exposure to PM_2.5_. There was also effect modification by hypertensive status for exposure to PM_10_ across the entire pregnancy, during the first trimester and during the second trimester with hypertensive women consistently having lower odds of preterm birth than normotensive women.

For the earliest preterm births (20–27 weeks gestation), among women without a hypertensive disorder, there were slightly higher odds for higher exposure to CO and PM_10_ and higher odds for exposure to NO_2_ and PM_2.5_ over the entire pregnancy. Patterns were similar among women with a hypertensive disorder and there was no significant effect modification.

There were no substantial differences after exclusion of women with diabetes or after adjustment for season of conception. When the sample was restricted to spontaneous preterm birth, many of the previous estimates were no longer statistically significant, probably due to the smaller sample size (results not shown).

## Discussion

This study aimed to disentangle the complex relationship among exposure to air pollution, maternal hypertensive disorders, and preterm birth. After stratification by maternal hypertensive disorders, associations between exposure to air pollution and preterm birth were variable. In general, there were moderately higher odds of preterm birth after exposure to air pollution among women without hypertension. Overall, the patterns were similar for women with a hypertensive disorder.

There were a few instances of statistically significant effect modification. However, most estimates were not meaningfully different between women with and without a hypertensive disorder. For those that were different such as the estimates for PM_10_ among births between 28 and 31 weeks, the estimates suggest an increased risk among women without a hypertensive disorder and no association or lower odds among those with a hypertensive disorder. There were a few exceptions like the first trimester exposures for late preterm births, but overall, the results were counter to our hypothesis. This analysis also included many comparisons and thus it is possible that some of the observed associations were due to chance.

There were also a few inverse associations and they tended to be for exposure at the end of pregnancy and stronger among women with a hypertensive disorder with a few exceptions. One could posit that these results could be attributed to unobserved fetal loss earlier in gestation. One prospective study observed faster time to fetal loss with higher exposure to PM_2.5_^13^ and another observed lower fecundability among couples exposed to higher levels of PM_2.5_.^14^ It is possible that women with high levels of exposure to air pollutants experienced fetal loss before a preterm birth could be observed or that especially sensitive women were unable to become pregnant. This may also be more likely among women with a hypertensive disorder. Some studies on pollution and fertility or miscarriage have observed similar results and some have not^15^ but these studies did not explore the potential differences by comorbid status.

Mechanistically, we hypothesized that the effect of exposure to air pollution might be modified by presence of a hypertensive disorder. Thus, we also explored the potential for hypertension to act as a mediator between air pollution and preterm birth. However, we did not observe a meaningfully higher odds of pregnancy-induced hypertension with higher exposure to air pollutants in this sample and thus, were not able to perform a full mediation analysis. This association has been previously observed^3^ and further investigation into mediation is warranted.

Our large, population-based sample came from a diverse area of California with high levels of pollution. Residences of our maternal participants were geocoded which allowed for very detailed air pollution exposure classification at specific time periods in gestation. The study was not designed to assess maternal comorbidities and characterization of hypertensive disorders relied on administrative data. OSHPD is a valuable source of information but sensitivity of administrative data and recording errors are a potential concern. A validation study of maternal conditions and obstetric conditions in California discharge data found diagnoses of preeclampsia to have a sensitivity and positive predictive value of about 80% but found diagnoses of hypertension to have a sensitivity of only about 60% and positive predictive value of 75%.^16^ However, underreporting of hypertension or preeclampsia is unlikely to be related to air pollution exposure and thus misclassification should be non-differential, resulting in attenuation of the estimates. Data on other potential confounders such as smoking and obesity were also not available or adequately collected and thus could not be adjusted for in this analysis.

This study adds some additional evidence to the hypothesis that exposure to higher levels of air pollution, especially particulate matter, is associated with preterm birth. There was some evidence of effect modification by maternal hypertensive status for exposure to PM_10_ for early preterm birth, but overall, hypertension did not modify the relationship between pollution and preterm birth. The inverse associations observed, especially among women with a hypertensive disorder, warrant further research into the potential effects of air pollution on early fetal loss to fully elucidate the mechanism by which air pollution influences preterm birth.

## Conflicts of interest statement

The authors declare that they have no conflicts of interest with regard to the content of this report.

Supported by F32HD096754 to investigator K.A.W from the Eunice Kennedy Shriver National Institute of Child Health & Human Development. This work was also supported by R21 ESO14891, P20 ES018173, P01ES022849, and R00ES021470 from the National Institute of Environmental Health Science, and the March of Dimes Prematurity Research Center at Stanford University. This publication was made possible by US Environmental Protection Agency STAR Grant RD83459601 and RD83543501. Its contents are solely the responsibility of the authors and do not necessarily represent the official views of the National Institutes of Health or the US EPA. Further, the US EPA does not endorse the purchase of any commercial products or services mentioned in the publication.

The data are publicly available from the Office of Statewide Health Planning and Development (OSHPD). The data are not available for replication because specific approvals from OSHPD and the California Committee for the Protection of Human Subjects must be obtained in order to access them.

## ACKNOWLEDGMENTS

We thank Bryan Penfold of Sonoma Technology, Inc. for traffic data processing and traffic density estimation.

## Supplementary Material
