# Supplementary material for: Air pollution, maternal hypertensive disorders, and preterm birth
Source: Environ Epidemiol. 2019 Aug 29;3(5):e062. doi: 10.1097/EE9.0000000000000062 (PMC7015251; doi:10.1097/EE9.0000000000000062)
Supplement: Supplementary file 1 [file ee9-3-e062-s001.docx]

| Supplemental Table 1. Distribution of pollutants averaged across the entire pregnancy | | | | |
| --- | --- | --- | --- | --- |
|  | Minimum | Median | Maximum | IQR* |
| CO (ppm) | 0.15 | 0.49 | 1.5 | 0.18 |
| NO_2_ (ppb) | 6.83 | 17.26 | 32.25 | 4.14 |
| PM_10_ (µg/m^3^) | 4.96 | 35.93 | 77.81 | 11.55 |
| PM_2.5_ (µg/m^3^) | 3.39 | 17.05 | 50.04 | 6.39 |
| Traffic density (300m) | 0 | 16.51 | 554.47 | 45.23 |
| *Interquartile range (25^th^ – 75^th^ percentiles) | | | | |

| Supplemental Table 2. Unadjusted associations between air pollutants and preterm birth by gestational age and timing of exposure, and effect modification by the presence of maternal hypertension, 2000-2006. | | | | | |
| --- | --- | --- | --- | --- | --- |
| Gestational Age | Pollutant Exposure | Exposure period | Odds Ratio  (95% Confidence Intervals) | | P-value interaction ^b^ |
|  |  |  | With Hypertension^a^ | Without Hypertension |  |
| 34-36 weeks | CO | Entire pregnancy | 1.03 (0.92-1.16) | **1.13 (1.09-1.18)** | 0.13 |
|  |  | 1^st^ trimester | **1.12 (1.01-1.24)** | **1.05 (1.01-1.08)** |  |
|  |  | 2^nd^ trimester | 0.99 (0.89-1.10) | 1.02 (0.98-1.05) |  |
|  |  | 3^rd^ trimester | 0.91 (0.81-1.01) | 0.99 (0.95-1.03) | 0.14 |
|  |  | Last 6 weeks | **0.86 (0.77-0.96)** | 0.98 (0.94-1.01) | 0.03 |
|  | NO_2_ | Entire pregnancy | 1.05 (0.94-1.17) | **1.10 (1.06-1.14)** |  |
|  |  | 1^st^ trimester | 1.06 (0.96-1.17) | **1.04 (1.01-1.07)** |  |
|  |  | 2^nd^ trimester | 0.96 (0.87-1.06) | **1.04 (1.01-1.08)** | 0.13 |
|  |  | 3^rd^ trimester | 0.96 (0.87-1.06) | 1.02 (0.99-1.05) |  |
|  |  | Last 6 weeks | 0.94 (0.84-1.04) | 1.02 (0.99-1.05) | 0.12 |
|  | PM_10_ | Entire pregnancy | 1.12 (1.00-1.24) | **1.09 (1.05-1.13)** |  |
|  |  | 1^st^ trimester | 1.10 (0.99-1.22) | **1.10 (1.06-1.13)** |  |
|  |  | 2^nd^ trimester | 1.05 (0.95-1.17) | **1.09 (1.05-1.13)** |  |
|  |  | 3^rd^ trimester | 0.99 (0.89-1.09) | **1.06 (1.02-1.09)** |  |
|  |  | Last 6 weeks | 1.01 (0.91-1.12) | 0.98 (0.95-1.02) |  |
|  | PM_2.5_ | Entire pregnancy | 1.11 (1.00-1.24) | **1.24 (1.20-1.28)** | 0.05 |
|  |  | 1^st^ trimester | 1.10 (0.99-1.21) | **1.04 (1.01-1.07)** |  |
|  |  | 2^nd^ trimester | 1.02 (0.92-1.12) | **1.03 (1.00-1.07)** |  |
|  |  | 3^rd^ trimester | **0.87 (0.78-0.96)** | **0.94 (0.91-0.97)** | 0.17 |
|  |  | Last 6 weeks | **0.89 (0.80-0.99)** | 0.99 (0.96-1.03) | 0.06 |
|  | Traffic Density | Entire pregnancy | 1.10 (0.99-1.22) | **1.09 (1.05-1.13)** |  |
| 32-33 weeks | CO | Entire pregnancy | 1.11 (0.91-1.36) | **1.19 (1.09-1.29)** |  |
|  |  | 1^st^ trimester | 1.15 (0.95-1.39) | 0.94 (0.87-1.02) | 0.06 |
|  |  | 2^nd^ trimester | 0.96 (0.79-1.17) | 0.98 (0.90-1.07) |  |
|  |  | 3^rd^ trimester | 0.88 (0.72-1.08) | 1.07 (0.98-1.16) | 0.09 |
|  |  | Last 6 weeks | 0.89 (0.73-1.09) | 1.06 (0.97-1.15) | 0.1 |
|  | NO_2_ | Entire pregnancy | 1.17 (0.97-1.42) | **1.16 (1.07-1.26)** |  |
|  |  | 1^st^ trimester | **1.22 (1.03-1.46)** | 1.01 (0.93-1.08) | 0.04 |
|  |  | 2^nd^ trimester | 1.00 (0.83-1.20) | 1.07 (0.99-1.15) |  |
|  |  | 3^rd^ trimester | 0.93 (0.77-1.12) | **1.09 (1.01-1.18)** | 0.1 |
|  |  | Last 6 weeks | 0.90 (0.75-1.09) | 1.06 (0.98-1.15) | 0.1 |
|  | PM_10_ | Entire pregnancy | 1.06 (0.87-1.30) | **1.15 (1.06-1.25)** |  |
|  |  | 1^st^ trimester | 1.12 (0.93-1.35) | **1.19 (1.10-1.29)** |  |
|  |  | 2^nd^ trimester | 1.05 (0.87-1.27) | **1.13 (1.04-1.22)** |  |
|  |  | 3^rd^ trimester | 1.01 (0.84-1.22) | **1.12 (1.04-1.21)** |  |
|  |  | Last 6 weeks | 1.02 (0.85-1.23) | 1.02 (0.95-1.11) |  |
|  | PM_2.5_ | Entire pregnancy | **1.41 (1.18-1.70)** | **1.46 (1.35-1.58)** |  |
|  |  | 1^st^ trimester | 1.19 (1.00-1.42) | 0.99 (0.92-1.07) | 0.06 |
|  |  | 2^nd^ trimester | 1.02 (0.85-1.22) | 1.06 (0.98-1.14) |  |
|  |  | 3^rd^ trimester | **0.77 (0.63-0.94)** | 0.96 (0.89-1.04) | 0.04 |
|  |  | Last 6 weeks | 0.87 (0.72-1.06) | **1.09 (1.01-1.18)** | 0.03 |
|  | Traffic Density | Entire pregnancy | **1.29 (1.07-1.55)** | **1.16 (1.07-1.26)** |  |
| 28-31 weeks | CO | Entire pregnancy | 0.91 (0.70-1.19) | **1.24 (1.12-1.37)** | 0.03 |
|  |  | 1^st^ trimester | 0.86 (0.67-1.10) | 1.04 (0.95-1.15) | 0.16 |
|  |  | 2^nd^ trimester | 0.82 (0.64-1.05) | 1.03 (0.93-1.13) | 0.1 |
|  |  | 3^rd^ trimester | 0.89 (0.69-1.16) | 1.01 (0.91-1.13) |  |
|  |  | Last 6 weeks | 0.85 (0.66-1.09) | 1.03 (0.93-1.14) | 0.16 |
|  | NO_2_ | Entire pregnancy | 1.00 (0.79-1.28) | **1.23 (1.12-1.35)** | 0.12 |
|  |  | 1^st^ trimester | 0.98 (0.79-1.23) | **1.20 (1.10-1.31)** | 0.1 |
|  |  | 2^nd^ trimester | 0.85 (0.68-1.07) | 1.07 (0.98-1.18) | 0.06 |
|  |  | 3^rd^ trimester | 0.92 (0.72-1.17) | 1.01 (0.92-1.11) |  |
|  |  | Last 6 weeks | 0.88 (0.70-1.12) | 1.05 (0.96-1.15) | 0.18 |
|  | PM_10_ | Entire pregnancy | **0.52 (0.38-0.71)** | **1.12 (1.01-1.24)** | < 0.001 |
|  |  | 1^st^ trimester | 0.78 (0.61-1.01) | **1.17 (1.07-1.29)** | 0.003 |
|  |  | 2^nd^ trimester | **0.74 (0.57-0.96)** | 1.09 (0.99-1.20) | 0.005 |
|  |  | 3^rd^ trimester | 0.86 (0.67-1.11) | 0.96 (0.86-1.06) |  |
|  |  | Last 6 weeks | 0.81 (0.63-1.03) | 0.97 (0.88-1.07) | 0.16 |
|  | PM_2.5_ | Entire pregnancy | 1.16 (0.92-1.46) | **1.39 (1.27-1.53)** | 0.15 |
|  |  | 1^st^ trimester | 0.98 (0.79-1.22) | 1.08 (0.99-1.18) |  |
|  |  | 2^nd^ trimester | 0.93 (0.74-1.17) | 1.06 (0.97-1.16) |  |
|  |  | 3^rd^ trimester | 0.81 (0.63-1.04) | 0.92 (0.83-1.02) |  |
|  |  | Last 6 weeks | 0.95 (0.75-1.20) | 1.07 (0.97-1.17) |  |
|  | Traffic Density | Entire pregnancy | 0.90 (0.70-1.15) | **1.13 (1.03-1.25)** | 0.08 |
| 20-27 weeks | CO | Entire pregnancy | 1.39 (0.90-2.14) | 1.12 (0.97-1.29) |  |
|  |  | 1^st^ trimester | 1.13 (0.74-1.73) | 1.03 (0.91-1.18) |  |
|  |  | 2^nd^ trimester | 1.06 (0.69-1.63) | 1.02 (0.89-1.16) |  |
|  |  | 3^rd^ trimester | N/A | N/A |  |
|  |  | Last 6 weeks | 1.18 (0.77-1.81) | 1.04 (0.91-1.20) |  |
|  | NO_2_ | Entire pregnancy | **1.57 (1.06-2.32)** | **1.29 (1.14-1.46)** |  |
|  |  | 1^st^ trimester | 1.33 (0.91-1.95) | 1.08 (0.96-1.22) |  |
|  |  | 2^nd^ trimester | 0.77 (0.51-1.18) | 1.09 (0.96-1.23) | 0.13 |
|  |  | 3^rd^ trimester | N/A | N/A |  |
|  |  | Last 6 weeks | 0.91 (0.61-1.38) | 1.11 (0.98-1.26) |  |
|  | PM_10_ | Entire pregnancy | 1.00 (0.65-1.54) | **1.18 (1.03-1.34)** |  |
|  |  | 1^st^ trimester | 1.35 (0.92-2.00) | **1.17 (1.03-1.32)** |  |
|  |  | 2^nd^ trimester | 1.07 (0.71-1.61) | 1.03 (0.91-1.17) |  |
|  |  | 3^rd^ trimester | N/A | N/A |  |
|  |  | Last 6 weeks | 0.97 (0.64-1.46) | **0.87 (0.76-0.99)** |  |
|  | PM_2.5_ | Entire pregnancy | **1.76 (1.20-2.59)** | **1.58 (1.40-1.78)** |  |
|  |  | 1^st^ trimester | 1.30 (0.89-1.91) | 1.06 (0.94-1.20) |  |
|  |  | 2^nd^ trimester | 0.99 (0.67-1.48) | 1.05 (0.93-1.19) |  |
|  |  | 3^rd^ trimester | N/A | N/A |  |
|  |  | Last 6 weeks | 1.16 (0.78-1.72) | 1.07 (0.94-1.21) |  |
|  | Traffic Density | Entire pregnancy | 1.27 (0.85-1.89) | 1.12 (0.98-1.27) |  |
| ^a^Pregnancy-induced and pre-pregnancy hypertension  ^b^P-values <0.2 for Wald’s Chi-squared test for interaction are reported | | | | | |
